# Burkitt's Lymphoma of the Gastrohepatic Omentum: A Malignant Presentation of Pseudoachalasia

**DOI:** 10.1155/2019/1803036

**Published:** 2019-01-13

**Authors:** Eric Omar Then, Andrew Ofosu, Prashanth Rawla, Tagore Sunkara, Sriharsha Dadana, Andrea Culliford, Vinaya Gaduputi

**Affiliations:** ^1^Division of Gastroenterology and Hepatology, SBH Health System, 4422 Third Ave, Bronx, New York 10457, USA; ^2^Division of Gastroenterology and Hepatology, The Brooklyn Hospital Center, Clinical Affiliate of The Mount Sinai Hospital, 121 Dekalb Ave, Brooklyn, NY 11201, USA; ^3^Department of Internal Medicine, Memorial Hospital of Martinsville and Henry County, 320 Hospital Drive Martinsville, VA 24115, USA; ^4^Division of Gastroenterology and Hepatology, Mercy Medical Center, 111 6th avenue Des Moines, IA 50314, USA

## Abstract

Achalasia is an intrinsic disorder of the esophagus that results from loss of ganglion cells in the lower esophageal sphincter. Clinically it is manifested by dysphagia to solids and liquids, weight loss, regurgitation, and chest pain. Pseudoachalasia, in contrast, is a rare entity that causes identical symptoms, but has a divergent underlying pathogenesis. The symptomology in these cases oftentimes occurs secondary to extrinsic compression of the esophagus, mostly attributable to malignancy. Although many cases of extrinsic esophageal compression have been reported in the literature, rarely has this occurred secondary to Burkitt's lymphoma in an adult. Here, we present a case of Burkitt's lymphoma resulting in pseudoachalasia in a 70-year-old female. The concurrence of these two entities in one patient makes this case presentation especially rare.

## 1. Introduction

First described by Ogilvie in 1947, pseudoachalasia is a rare secondary cause of achalasia that is not attributable to intrinsic esophageal disease [1]. To put its rarity into context, the incidence rate of achalasia in the general population is 1 in 100,000 people worldwide [2]. Pseudoachalasia is thought to account for up to 4% of these cases [3]. Burkitt's lymphoma (BL), a disease most often seen in African children, is an even more rare entity when seen in adults in the Americas. The incidence rate of sporadic type BL, the variant most often seen in the United States, is 3 cases per million in both children and adults [4].

## 2. Case

**Figure 1:**
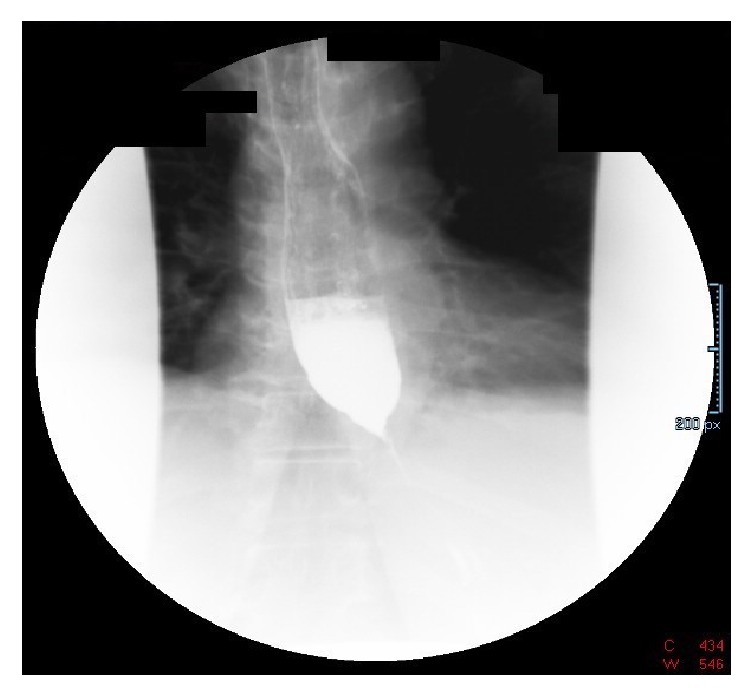
Barium swallow showing classic bird beak configuration consistent with achalasia.

**Figure 2:**
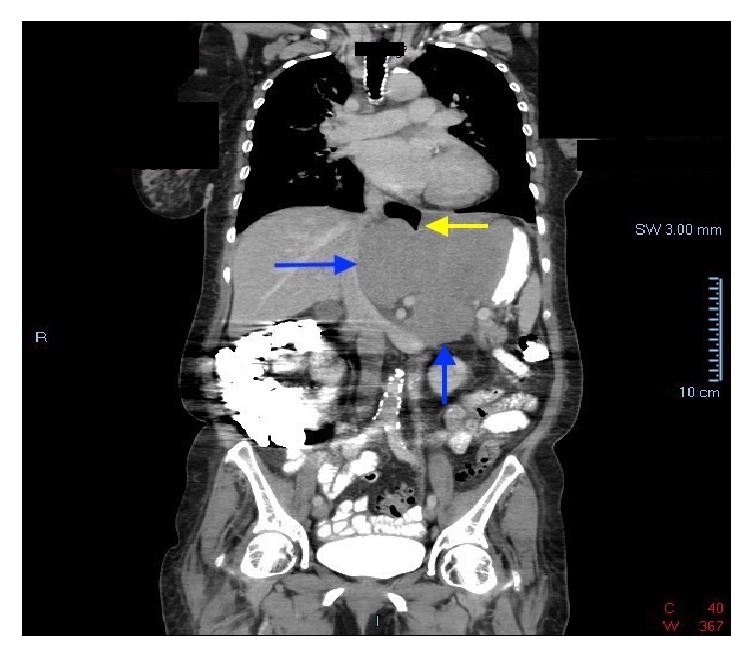
CT scan (coronal view) showing a 12 x 12 soft tissue mass in the gastrohepatic omentum (blue arrows) compressing the distal esophagus (yellow arrow) and gastric fundus.

**Figure 3:**
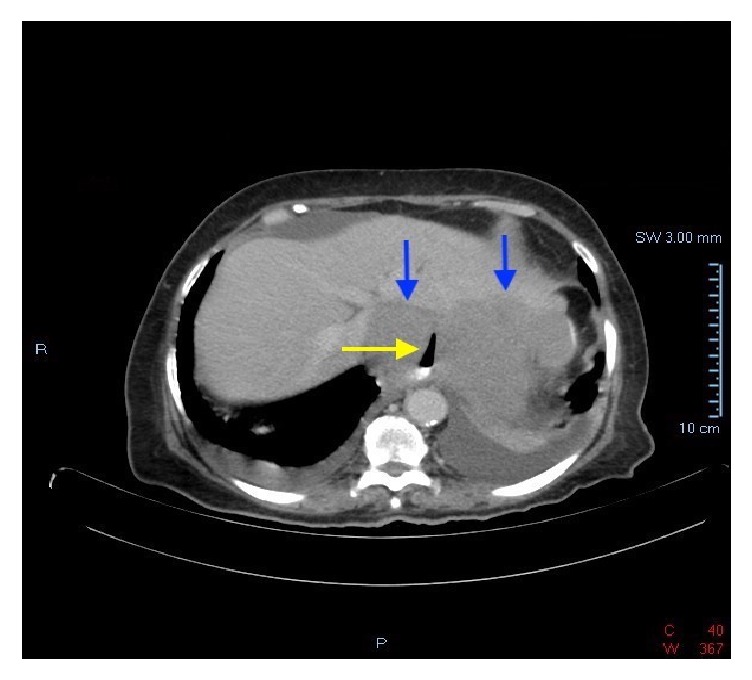
CT scan (axial view) showing a 12 x 12 soft tissue mass in the gastrohepatic omentum (blue arrows) compressing the distal esophagus (yellow arrow) and gastric fundus.

**Figure 4:**
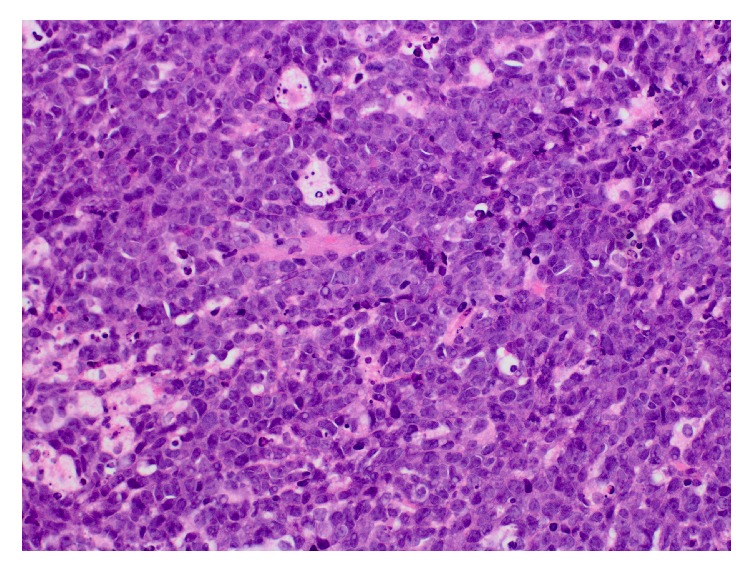
Biopsy (hematoxylin and eosin stain) showing a classic “starry sky” appearance consistent with Burkitt's lymphoma.

**Figure 5:**
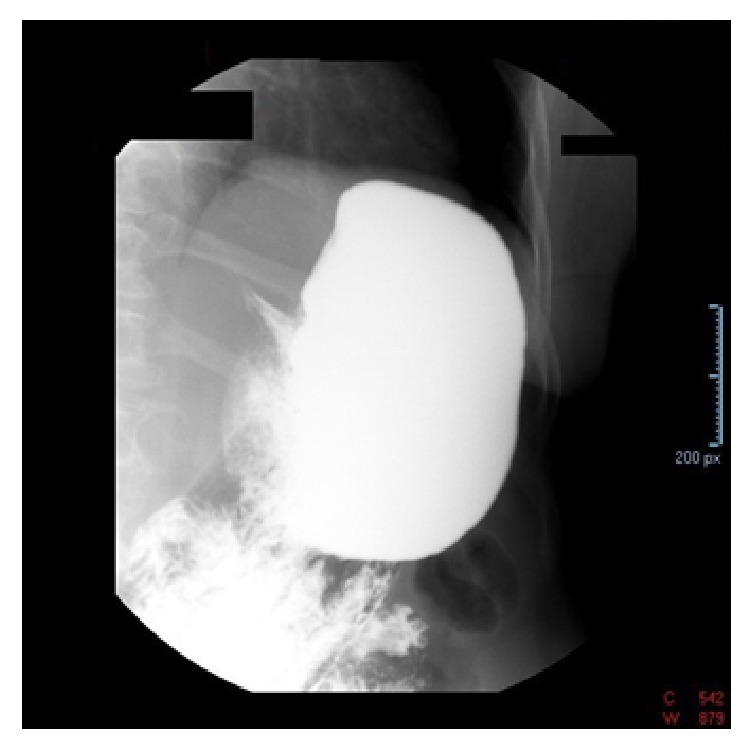
Barium swallow showing resolution of bird beak appearance with complete filling of the stomach.

Patient is a 70-year-old female with a past medical history of diabetes mellitus, hyperlipidemia, fibromyalgia, rheumatoid arthritis, and depression, who was referred to our institution's gastroenterology clinic for dysphagia to solids for 1 month. This was accompanied by 2-3 episodes of vomiting daily and a 30-pound weight loss. Due to her alarm symptoms an upper endoscopy was scheduled. The upper endoscopy revealed luminal narrowing in the lower 1/3 of the esophagus without any discernable esophageal web or ring. During the procedure, intubation of the stomach was difficult, but showed nonbleeding erosive antral gastropathy. On follow-up appointment 6 days later, the patient reported progression of symptoms, now complaining of dysphagia to liquids. The patient was then referred to the emergency department due to inability to tolerate oral intake. On admission workup included a barium swallow showing abrupt tapering of the gastroesophageal junction with a bird beak configuration consistent with achalasia (Figure 1). These radiologic findings, coupled with her symptoms, raised our suspicion of intrinsic achalasia as the culprit. The patient was then taken to the endoscopy suite to undergo palliative treatment with a botulinum injection to the lower esophageal sphincter. One day later, however, the patient's symptoms showed no improvement. Given her lack of clinical improvement, the differential diagnosis now included pseudoachalasia as a possible cause. A CT scan of the chest and abdomen was then done to rule out extrinsic compression of the esophagus. This showed a 12 x 12 soft tissue mass in the gastrohepatic omentum compressing the distal esophagus and gastric fundus (Figures 2 and 3). Due to the size of the mass and the small sample size that would have been obtained with FNA, EUS was not done. Instead a CT guided biopsy was done, the results of which showed a classic “starry sky” appearance consistent with Burkitt's lymphoma (Figure 4). After tissue diagnosis, the patient was transferred to an outside institution to undergo chemotherapy. During her course at our institution, her nutritional needs were met through total parenteral nutrition. 3 months later she presented to our gastroenterology clinic for follow-up with complete resolution of symptoms. Repeat barium swallow was done showing resolution of the previously seen birds beak appearance with complete esophagogastric emptying (Figure 5).

## 3. Discussion

Several entities have been linked to the development of pseudoachalasia. These include malignancy (primary and metastatic), benign conditions (Tuberculosis, Sarcoidosis, amyloidosis, pancreatic pseudocysts), paraneoplastic syndromes, and postoperative sequelae (bariatric banding, vagotomy, Nissen fundoplication) [5–7]. Other less common causes that have been reported in the literature include CNS disorders (Arnold-Chiari malformations, meningomyeloceles) and thoracic aortic aneurysms [8, 9]. Of the aforementioned entities, malignancy of the esophagus and gastric cardia are the most common culprits in up to 70% of cases [10]. With that being said, to our knowledge our case is the first of its kind, with pseudoachalasia occurring secondary to extrinsic esophageal compression from BL.

Signs and symptoms of pseudoachalasia are nearly identical to those of achalasia. Progressive dysphagia to solids and liquids is the most predominant symptom that occurs in both. This is usually accompanied by a corresponding weight loss due to decreased caloric intake. Clues that should steer the clinician to suspect pseudoachalasia, rather than achalasia, are the duration of symptoms and amount of weight lost in that period of time. A literature review conducted by Kahrilas et al. showed that patients with achalasia experienced symptoms for 24 months, with a median weight loss of 5 pounds. In contrast, patients with pseudoachalasia experienced symptoms for 3 months, with a median weight loss of 22 pounds [11]. Awareness of these differences is crucial as prompt recognition can translate into quicker diagnosis of a possible underlying malignancy.

Present day, the gold standard for diagnosing achalasia is esophageal manometry. In many cases, however, this modality is unable to distinguish achalasia from pseudoachalasia. Both diseases will demonstrate lack of peristalsis and incomplete relaxation of the lower esophageal sphincter (LES) [8]. Endoscopy, the first modality employed after a patient presents with dysphagia, can provide subtle differences between the two. In patients with pseudoachalasia, passage of the endoscope through the LES requires more pressure than in patients with true achalasia [12]. In cases where the clinical picture suggests pseudoachalasia, but endoscopy and manometry are ambiguous, endoscopic ultrasound (EUS) and CT scan are useful alternatives. CT scans showing esophageal wall thickening, soft tissue masses at the esophageal-gastric junction, mediastinal lymphadenopathy, and pulmonary, hepatic, or osseous metastasis all suggest the presence of pseudoachalasia [13]. EUS is an effective modality in identifying level of tumor depth and invasion and in some cases allows one to obtain a biopsy through FNA [14, 15]. In our case, CT scan was able to elucidate the cause of the achalasia, revealing a soft tissue mass obstructing the esophagus, which was later diagnosed as BL.

Given the varying etiologies of pseudoachalasia, treatment of this entity is contingent on the underlying pathologic process. In patients where malignancy is the culprit, radiotherapy and chemotherapy have been shown to relieve esophageal obstruction [16, 17]. Botulinum injection is an effective option to improve symptoms in cases secondary to amyloidosis and Sarcoidosis [18, 19]. Cases that are refractory to conservative therapy or arise as a complication of fundoplication require surgical treatment with Heller myotomy [8, 20]. Currently treatment with self-expandable metal stents (SEMS) is becoming a more popular option in patients seeking more rapid palliation of symptoms. Whereas chemotherapy and radiotherapy take several weeks to improve dysphagia, treatment with SEMS provides immediate relief [21]. Although it has been associated with complications such as perforation, bleeding, pain, and fistula formation, it has better long-term outcomes than pneumatic dilation [22].

## 4. Conclusion

In conclusion pseudoachalasia is a rare manifestation of achalasia that can oftentimes be a challenge to diagnose. Due to their similar clinical presentation, it is important to make the distinction between these two entities given the potential for underlying malignancy. Once diagnosed, therapy should be tailored to palliate symptoms whilst targeting the underlying disease process.
